# Impaired mitochondrial function abolishes gamma oscillations in the hippocampus through an effect on fast-spiking interneurons

**DOI:** 10.1093/brain/awr018

**Published:** 2011-03-04

**Authors:** Roger G. Whittaker, Douglass M. Turnbull, Miles A. Whittington, Mark O. Cunningham

**Affiliations:** 1 Institute of Neuroscience, Newcastle University, Newcastle upon Tyne, NE1 7RU, UK; 2 Institute for Ageing and Health, Newcastle University, Newcastle upon Tyne, NE4 5PL, UK

Sir, Synchronous neuronal oscillations in the gamma frequency band (30–80  Hz) are implicated in a wide range of cognitive processes, including memory formation (Varela *et al*., 2001) and sensory processing (Singer, 1993; Gray, 1994). This rapid, temporally co-ordinated activity in spatially distributed areas of cortex is highly energy dependent, producing large changes in blood-oxygen level dependent signals *in vivo* and showing exquisite sensitivity to hypoxia *in vitro* (Huchzermeyer, 2008). Gamma oscillations are generated by reciprocal excitation and inhibition in networks of electrically coupled pyramidal cells and GABAergic interneurons (Fisahn *et al*., 1998; Cunningham *et al*., 2003; Hájos *et al*., 2004). Synchronous firing of networks of basket-cell interneurons at gamma frequency produces phasic inhibition of pyramidal cells, the periods when this inhibition is minimal, providing temporal windows for pyramidal cell firing. Pyramidal cell firing in turn provides reciprocal tonic excitation onto interneurons, allowing the cycle to continue (Traub *et al*., 1996). The research article by Kann *et al*. (2011) demonstrates the critical dependence of gamma oscillations upon mitochondrial function. However, little is known about the relative energetic requirements of the different cellular components of this network. We have addressed this issue with intracellular recordings of pyramidal cells and interneurons undergoing gamma oscillations under conditions of metabolic stress *in vitro*.

Rat hippocampal slices (450 μm horizontal sections) were maintained in a recording chamber at the interface between 95% oxygen/5% CO_2_ and artificial CSF (in mM: 10 glucose, 126 NaCl, 3 KCl, 24 NaHCO_3_, 1.25 NaH_2_PO_4_, 2 MgSO_4_ and 2 CaCl_2_). Stable gamma oscillation was induced by adding low dose (100  nM) kainic acid to the perfusate. Local field potential recordings were made from the stratum pyramidale layer in CA3 region of the hippocampus using glass microelectrodes (10–300  Hz band-pass filtered, digitized at 10  kHz). We also performed simultaneous intracellular recordings from CA3 pyramidal cells using sharp microelectrodes (70–130  MΩ) containing 2  M potassium acetate. Cells were recorded in current clamp mode, and identified electrophysiologically on the basis of their low resting firing rate, accommodating response to sustained depolarization and gamma frequency phasic inhibitory post-synaptic potentials. Excitatory post-synaptic potentials (EPSPs) and inhibitory post-synaptic potentials (IPSPs) were recorded at −70 and −20  mV, respectively.

We then measured the effect on gamma oscillation power and corresponding intracellular activity using a variety of mitochondrial respiratory chain inhibitors. Potassium cyanide (KCN; 100  μM), an inhibitor of cytochrome oxidase in complex IV of the mitochondrial respiratory chain, caused a 72.1% reduction in gamma power (1221.3 ± 207.8  μV^2^ control versus 340.8 ± 70.5  μV^2^ KCN, *n* = 9, *P* < 0.05; Fig. 1A(i)) within 15  min. There was no significant change in the resting membrane potential (−57.9 ± 1.29  mV versus −60.6 ± 2.05  mV KCN, *P* > 0.05; Fig. 1A(ii)) of pyramidal cells. Despite this, there was a marked reduction in pyramidal cell firing rate during the concurrently recorded gamma frequency oscillation (3.02 ± 0.70  Hz versus 1.43 ± 0.64  Hz KCN, *n* = 9, *P* < 0.05). We therefore examined the synaptic inputs to pyramidal cells. There was no significant change in excitatory input (mean EPSP amplitude 3.18 ± 0.70  mV control versus 2.43 ± 0.72  mV KCN, *P* > 0.05; mean EPSP frequency 12.4 ± 2.6  Hz control versus 11.9 ± 1.9  Hz KCN, *P* > 0.05). The mean IPSP amplitude was reduced, (6.80 ± 1.66  mV control versus 5.21 ± 0.74  mV KCN, *P* < 0.05); however, the most striking finding was a marked reduction in the frequency of IPSPs impinging on the pyramidal cells (32.4 ± 2.6  Hz control versus 26.2 ± 3.9  Hz KCN, *P* < 0.05; Fig. 1A(iv)). We found similar effects on pyramidal cell firing and synaptic inputs using 1  μM Rotenone, an inhibitor of complex I, and 1  μM FCCP, a protonophore that discharges the mitochondrial membrane electrochemical gradient. With all three inhibitors, gamma power and pyramidal cell function returned to control levels on washing out of the inhibitors.

**Figure 1:**
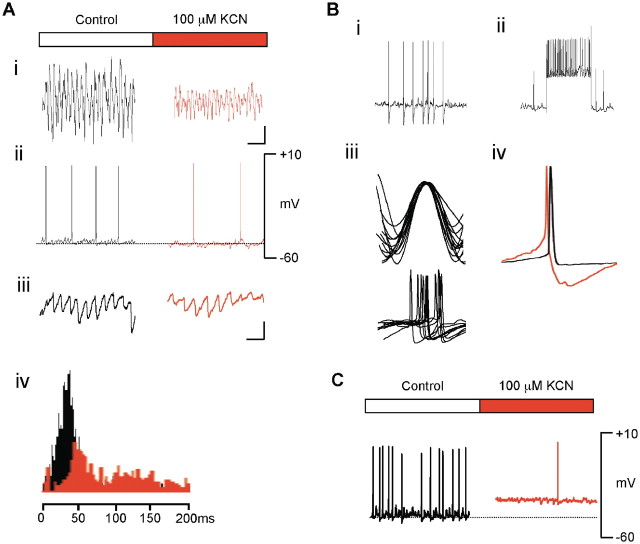
(**A**) (i) Local field potential recording, CA3 region of hippocampus. Low dose (100 μM) KCN significantly reduces gamma oscillation power. Scale bar: horizontal 100  ms, vertical 200  μV. (ii) Simultaneous intracellular recording, CA3 stratum pyramidale pyramidal cell. KCN causes reduced resting firing rate with no significant change in resting membrane potential. (iii) Inhibitory post-synaptic potentials recorded at −20  mV. Scale bar: horizontal 50  ms, vertical 2  mV. (iv) Inter-event intervals of IPSPs impinging on pyramidal neuron. Mean frequency of IPSPs declines significantly. (**B**) (i) CA3 stratum pyramidale interneurons show rapid resting firing rates, (ii) show little accommodation to sustained depolarization, (iii) fire in phase with gamma field potential and, (iv) show prominent after-hyperpolarization potentials (pyramidal cell, black; interneuron, red). (**C**) Addition of KCN causes sustained depolarization of CA3 interneurons with marked reduction in resting firing rate.

Having demonstrated a predominant effect on the inhibitory input to pyramidal cells, we undertook intracellular recordings from CA3 stratum pyramidale interneurons (*n* = 3) that contribute to this input. These were identified electrophysiologically as basket cells on the basis of high resting firing rates, lack of accommodation to sustained depolarization, prominent after-hyperpolarization potentials and firing in-phase with the gamma field oscillation. Addition of 100  μM KCN caused a sustained depolarization of the mean membrane potential recorded during gamma oscillations (−50.4 ± 5.37  mV control versus −37.2 ± 2.98  mV KCN, *P* < 0.05) and produced an almost complete abolition of firing (9.92 ± 1.75  Hz control versus 0.11 ± 0.07  Hz KCN, *P* < 0.05).

In agreement with the findings of Kann *et al*. (2011), we demonstrate the extreme sensitivity of gamma oscillations to metabolic stress, and in particular to disruption of either complex I or complex IV in the mitochondrial respiratory chain, or of the mitochondrial membrane potential. Gamma oscillations are dependent on inhibitory input to pyramidal cells, and although these cells receive input from many different sub-types of inhibitory interneurons, it is the input from fast-spiking basket cells that primarily determine the frequency and power of the resulting network oscillations (Fuchs *et al*., 2007; Middleton *et al*., 2008). Within this context, our results suggest that the decline in gamma oscillation power seen with inhibitors of the mitochondrial respiratory chain results primarily from effects on these fast spiking interneurons. Concentrations of inhibitor that caused no significant effect on pyramidal cell resting membrane potential, caused a marked and sustained membrane depolarization in fast-firing interneurons. This in turn led to an almost complete cessation of interneuron firing, presumably as a result of inactivation of voltage-gated sodium channels (Martin *et al*., 2010). This exquisite sensitivity of interneurons is not unexpected; basket cells are known to contain high concentrations of cytochrome oxidase *c* and greater numbers of mitochondria compared to pyramidal cells (Gulyás *et al*., 2006), suggesting a heavy reliance on respiratory chain function to maintain their rapid firing rates.

Gamma oscillations are emerging as a fundamental cortical network behaviour. Disruption of fast-spiking interneuron function is observed in a number of neuropsychiatric and neurodegenerative conditions such as schizophrenia, Parkinson’s and Alzheimer’s disease. Importantly, in all of these conditions, disturbances in gamma oscillations dynamics have been observed (Uhlhaas and Singer, 2006). Our findings may offer insight into pathophysiology of cortical dysfunction both in genetically determined diseases of the mitochondrial respiratory chain, but also in other neurodegenerative conditions in which secondary mitochondrial dysfunction is implicated (DiMauro and Schon, 2008).

## Funding

This work was supported by the European Union (EUmitocombat) and the Royal Society.
